# “Journal” Change

**Published:** 1896-01

**Authors:** 


### EDITORIAL.

### "JOURNAL" CHANGE.

WITH the December number Dr. W. A. Conklin severed his connection with the JOURNAL. Long identified with its publication, he was a liberal giver to the veterinary profession, and spent several thousands of dollars in the early history of the JOURNAL to make it worthy of a place among scientific publications. His pen for many years was kept busy in the interest of a higher education for the veterinarian, and the early history of this publication owes largely its sustenance to his indefatigable labors. Other interests the past few years have demanded his whole time and attention, and the greater needs of journalism to-day made greater the demands upon his time and money than he could well give. We will not be without his advice and aid when needed in the better direction of JOURNAL affairs, and from time to time we shall hope to have his pen wielded for the benefit of our readers.

